# Supplementary material for: Examining trends in substance use disorder capacity and service delivery by Health Resources and Services Administration-funded health centers: A time series regression analysis
Source: PLoS One. 2020 Nov 30;15(11):e0242407. doi: 10.1371/journal.pone.0242407 (PMC7703936; doi:10.1371/journal.pone.0242407)
Supplement: S1 Table — (DOCX) [file pone.0242407.s001.docx]

| S1 Table. Full regression models of substance use disorder capacity, service use, and panel size and visit ratio by grantee status | | | | | | | | | | | | | | |
| --- | --- | --- | --- | --- | --- | --- | --- | --- | --- | --- | --- | --- | --- | --- |
|  | **Proportion of HC with SUD personnel** | | **Number of SUD personnel at each HC** | | **SUD personnel per 1,000 patients at each HC** | | **Proportion of total HC patients that were SUD patients at each HC** | | **Proportion of total HC visits that were SUD visits at each HC** | | **SUD patients per SUD personnel at each HC** | | **SUD visit per SUD personnel at each HC** | |
| Sample size | 2,906 | | 2,906 | | 2,906 | | 2,809 | | 2,893 | | 1,063 | | 1,060 | |
|  | Coef. | 95% CI | Coef. | 95% CI | Coef. | 95% CI | Coef. | 95% CI | Coef. | 95% CI | Coef. | 95% CI | Coef. | 95% CI |
| Grant (ref. None) |  |  |  |  |  |  |  |  |  |  |  |  |  |  |
| SASE/AIMS | 5.117*** | [3.20,7.03] | 1.661*** | [1.02,2.30] | 1.005*** | [0.44,1.57] | 1.233** | [0.36,2.11] | 1.883*** | [0.93,2.83] | -0.934** | [-1.60,-0.27] | -0.742* | [-1.39,-0.09] |
| AIMS only | 1.737* | [0.08,3.40] | 0.508 | [-0.08,1.10] | 0.227 | [-0.26,0.72] | 0.322 | [-0.43,1.07] | 1.039* | [0.22,1.86] | -1.323*** | [-1.98,-0.67] | -0.671* | [-1.31,-0.04] |
| Year (ref. 2015) |  |  |  |  |  |  |  |  |  |  |  |  |  |  |
| 2016 | 0.235 | [-1.25,1.72] | -0.299 | [-0.81,0.21] | -0.218 | [-0.57,0.14] | -0.442*** | [-0.49,-0.39] | -0.184*** | [-0.20,-0.16] | 0.0155 | [-0.03,0.06] | 0.151*** | [0.13,0.17] |
| 2017 | -0.371 | [-1.92,1.17] | -0.0852 | [-0.57,0.40] | -0.175 | [-0.53,0.18] | -0.253*** | [-0.30,-0.20] | -0.00861 | [-0.03,0.01] | -0.570*** | [-0.62,-0.52] | -0.158*** | [-0.18,-0.13] |
| 2016*SASE/AIMS | 4.786*** | [3.01,6.56] | 0.555* | [0.02,1.09] | 0.419* | [0.04,0.80] | 0.504*** | [0.45,0.56] | 0.253*** | [0.23,0.27] | 0.0212 | [-0.03,0.07] | 0.376*** | [0.35,0.40] |
| 2017*SASE/AIMS | 6.673*** | [4.74,8.60] | 0.640* | [0.13,1.15] | 0.606** | [0.22,0.99] | 0.405*** | [0.35,0.46] | 0.175*** | [0.16,0.19] | 0.389*** | [0.34,0.44] | 0.632*** | [0.60,0.66] |
| 2016*AIMS only | 0.207 | [-1.34,1.76] | 0.387 | [-0.15,0.92] | 0.18 | [-0.20,0.56] | 0.441*** | [0.39,0.49] | 0.200*** | [0.18,0.22] | -0.031 | [-0.08,0.02] | 0.0224 | [-0.00,0.05] |
| 2017*AIMS only | 2.223** | [0.61,3.84] | 0.208 | [-0.30,0.72] | 0.116 | [-0.26,0.49] | 0.243*** | [0.19,0.29] | -0.131*** | [-0.15,-0.11] | 0.607*** | [0.55,0.66] | 0.279*** | [0.25,0.31] |
| *Health Center Characteristics* |  |  |  |  |  |  |  |  |  |  |  |  |  |  |
| Greater than 10,000 patients served | 0.148 | [-0.50,0.79] | 0.314* | [0.07,0.55] | -0.774*** | [-1.00,-0.55] | -0.276*** | [-0.31,-0.24] | 0.0272*** | [0.02,0.04] | -0.469*** | [-0.51,-0.42] | 0.118*** | [0.10,0.13] |
| Percent in Urban | -0.0906 | [-0.79,0.61] | 0.0952 | [-0.10,0.29] | 0.0644 | [-0.09,0.22] | 0.0314*** | [0.02,0.05] | -0.219*** | [-0.22,-0.21] | -0.0394*** | [-0.06,-0.02] | -0.328*** | [-0.34,-0.32] |
| Percent with Electronic Health Records | 0.289 | [-0.22,0.80] | -0.0954 | [-0.24,0.05] | -0.108 | [-0.23,0.02] | 0.00193 | [-0.01,0.02] | -0.0854*** | [-0.09,-0.08] | -0.117*** | [-0.13,-0.10] | -0.0941*** | [-0.10,-0.09] |
| Region (ref. Percent in New England) |  |  |  |  |  |  |  |  |  |  |  |  |  |  |
| Percent in Middle Atlantic | -1.525 | [-3.27,0.22] | -0.453 | [-1.11,0.21] | -0.14 | [-0.79,0.51] | 0.744 | [-0.31,1.80] | 0.148 | [-0.99,1.28] | 1.255*** | [0.73,1.78] | 0.611* | [0.11,1.11] |
| Percent in East North Central | -2.026* | [-3.67,-0.38] | -0.824** | [-1.42,-0.23] | -0.924** | [-1.51,-0.34] | -0.258 | [-1.23,0.71] | -0.911 | [-1.99,0.16] | 0.147 | [-0.33,0.63] | -0.561* | [-1.02,-0.10] |
| Percent in West North Central | -1.427 | [-3.40,0.55] | -0.962** | [-1.68,-0.24] | -1.202** | [-1.92,-0.48] | -0.761 | [-1.88,0.36] | -1.246* | [-2.48,-0.01] | 0.314 | [-0.30,0.93] | 0.0997 | [-0.50,0.70] |
| Percent in South Atlantic | -2.122* | [-3.79,-0.46] | -0.586 | [-1.18,0.01] | -0.601* | [-1.19,-0.02] | -0.271 | [-1.21,0.67] | -0.624 | [-1.66,0.41] | -0.03 | [-0.51,0.45] | -0.31 | [-0.77,0.15] |
| Percent in East South Central | -1.086 | [-3.03,0.85] | -1.037** | [-1.76,-0.32] | -1.573*** | [-2.30,-0.84] | -0.461 | [-1.60,0.68] | -0.91 | [-2.17,0.35] | 1.218*** | [0.61,1.82] | 0.371 | [-0.21,0.95] |
| Percent in West South Central | -2.247* | [-4.27,-0.22] | -1.227** | [-1.99,-0.46] | -1.167** | [-1.91,-0.43] | -1.126* | [-2.16,-0.09] | -1.05 | [-2.19,0.09] | 0.064 | [-0.54,0.67] | -0.778** | [-1.35,-0.20] |
| Percent in Mountain | -0.848 | [-2.67,0.98] | -0.689* | [-1.35,-0.03] | -0.926** | [-1.60,-0.25] | -0.41 | [-1.46,0.64] | -0.797 | [-1.96,0.37] | 0.0285 | [-0.51,0.56] | -0.518 | [-1.04,0.00] |
| Percent in Pacific | 0.226 | [-1.52,1.97] | 0.417 | [-0.20,1.04] | 0.25 | [-0.35,0.85] | 0.628 | [-0.30,1.56] | 0.355 | [-0.64,1.35] | -0.147 | [-0.58,0.29] | -0.714*** | [-1.13,-0.30] |
| *Health Center Patient Characteristics* |  |  |  |  |  |  |  |  |  |  |  |  |  |  |
| Percent of Patients that are Minority | 0.0143 | [-0.00,0.03] | 0.0114*** | [0.01,0.02] | 0.0122*** | [0.01,0.02] | -0.0116*** | [-0.01,-0.01] | 0.0138*** | [0.01,0.01] | -0.00229* | [-0.00,-0.00] | -0.00809*** | [-0.01,-0.01] |
| Percent of Patients that are Homeless | 0.0670*** | [0.05,0.09] | 0.00864** | [0.00,0.01] | 0.0166*** | [0.01,0.02] | 0.0115*** | [0.01,0.01] | -0.000864*** | [-0.00,-0.00] | 0.0127*** | [0.01,0.01] | 0.00287*** | [0.00,0.00] |
| Percent of Patients that are Migrant and Agricultural Workers | -0.0480* | [-0.09,-0.01] | -0.0280*** | [-0.04,-0.01] | -0.0456*** | [-0.06,-0.03] | 0.0646*** | [0.06,0.07] | 0.0408*** | [0.04,0.04] | 0.0578*** | [0.05,0.06] | 0.0326*** | [0.03,0.04] |
| Percent of Patients that are less than 100% of the Federal Poverty Guideline | 0.0173* | [0.00,0.03] | 0.00465* | [0.00,0.01] | 0.00359* | [0.00,0.01] | -0.00245*** | [-0.00,-0.00] | 0.00203*** | [0.00,0.00] | -0.00171*** | [-0.00,-0.00] | -0.00155*** | [-0.00,-0.00] |
| Percent of Patients that are Uninsured | 0.0238 | [-0.00,0.05] | -0.000924 | [-0.01,0.01] | 0.000256 | [-0.01,0.01] | -0.00510*** | [-0.01,-0.00] | -0.00718*** | [-0.01,-0.01] | 0.0118*** | [0.01,0.01] | 0.0259*** | [0.03,0.03] |
| Percent of Patients that are Medicaid | -0.00545 | [-0.03,0.02] | -0.00185 | [-0.01,0.01] | -0.00294 | [-0.01,0.01] | -0.0115*** | [-0.01,-0.01] | -0.00226*** | [-0.00,-0.00] | 0.00536*** | [0.00,0.01] | 0.0149*** | [0.01,0.02] |
| *Quality Indicators* |  |  |  |  |  |  |  |  |  |  |  |  |  |  |
| Percent with PCMH Recognition | 0.393 | [-0.21,0.99] | 0.197 | [-0.01,0.40] | 0.00563 | [-0.17,0.18] | 0.171*** | [0.15,0.19] | 0.0964*** | [0.09,0.10] | 0.0228 | [-0.00,0.05] | -0.117*** | [-0.13,-0.10] |
| *Substance Abuse Market Availability* |  |  |  |  |  |  |  |  |  |  |  |  |  |  |
| Mean number of facilities per 100,000 persons providing substance abuse services | 0.0923 | [-0.09,0.28] | 0.0571 | [-0.02,0.13] | 0.0970* | [0.02,0.18] | -0.0881*** | [-0.10,-0.07] | 0.0345*** | [0.03,0.04] | -0.0917*** | [-0.11,-0.07] | -0.0456*** | [-0.05,-0.04] |
| *Substance Abuse Need* |  |  |  |  |  |  |  |  |  |  |  |  |  |  |
| Opioid mortality rate per 100,000 persons | 0.0382 | [-0.01,0.09] | 0.00711 | [-0.01,0.02] | 0.00343 | [-0.01,0.02] | 0.0188*** | [0.02,0.02] | 0.00210*** | [0.00,0.00] | 0.00573*** | [0.00,0.01] | -0.0127*** | [-0.01,-0.01] |
| *Statistically significant at *p<0.05; **p<0.01; ***p<0.001* | | | | | | | | | | | | | | |
| *SUD, substance use disorder; AIMS, Access Increases in Mental Health and Substance Abuse Services; SASE, Substance Abuse Service Expansion; HC, health center; CI, confidence interval; PCMH, patient centered medical home.* | | | | | | | | | | | | | | |
