# Supplementary material for: Examining trends in substance use disorder capacity and service delivery by Health Resources and Services Administration-funded health centers: A time series regression analysis
Source: PLoS One. 2020 Nov 30;15(11):e0242407. doi: 10.1371/journal.pone.0242407 (PMC7703936; doi:10.1371/journal.pone.0242407)
Supplement: S2 Table — (DOCX) [file pone.0242407.s002.docx]

| **S2 Table. Percent change and predicted probabilities of substance use disorder patients and visits by grantee status, from 2010 to 2017** | | | | | | | | | | | | | | | | | | | |
| --- | --- | --- | --- | --- | --- | --- | --- | --- | --- | --- | --- | --- | --- | --- | --- | --- | --- | --- | --- |
|  | **None** | | | **Only AIMS^b^** | | | **SASE/AIMS^a,b^** | | | **Percent Change 2015 - 2017** | | | | | **Predicted Probability [95% CI]** | | | |  |
|  | **2015** | **2016** | **2017** | **2015** | **2016** | **2017** | **2015** | **2016** | **2017** | **None** | | **Only AIMS^b^** | **SASE/AIMS^a,b^** | | **AIMS only vs. none** | **SASE/AIMS vs. none** | | **SASE/AIMS vs AIMS only** |  |
| Sample of HCs | 13% (176) |  |  | 68% (907) |  |  | 19% (258) |  |  | 13% (176) | | 68% (907) | 19% (258) | | 13% (176) | 68% (907) | | 19% (258) |  |
|  | *Mean (SD)* | | | *Mean (SD)* | | | *Mean (SD)* | | |  |  | | |  |  | |  |  |  |
| ***SUD Service Use*** |  |  |  |  |  |  |  |  |  |  | |  |  | |  |  | |  |  |
| Average Number of Total SUD Patients | 142 (56) | 84 (33) | 111 (44) | 235 (53) | 251 (57) | 259 (58) | 902 (266) | 992 (293) | 1,150 (340) | -22% | | 10% | 27% | | 55 [25,85]*** | 279 [127,430]*** | | 224 [83,364]*** |  |
| Average Number of Total SUD Visits | 232 (95) | 197 (81) | 266 (109) | 1,284 (260) | 1,405 (284) | 1,223 (247) | 4,581 (1,573) | 5,281 (1,813) | 6,212 (2,133) | 15% | | -5% | 36% | | -96 [-131,-60]*** | 1,596 [497,2,695]*** | | 1,692 [587, 2797]*** |  |
| *^a^ HRSA distributed $94 million in March 2016 through the Substance Abuse Service Expansion (SASE) program to 271 HCs ($217,000 to $406,000) with the goals of increasing SUD personnel, increasing number of patients screened and connected to SUD treatment, and increasing access to Medication Assisted Treatment (MAT) services.* | | | | | | | | | | | | | | | | | | | |
| *^b^ HRSA distributed $200 million in September 2017 through the Access Increases in Mental Health and Substance Abuse Services (AIMS) program to 1,178 HCs ($84,000 to $176,000) with the goals of increasing substance abuse services focusing on the treatment, prevention, and awareness of opioid abuse; increasing SUD personnel; and leveraging health information technology and training to increase and improve SUD services.* | | | | | | | | | | | | | | | | | | | |
| *Standard deviation or count in parentheses.* | | | | | | | | | | | | | | | | | | | |
| *Statistically significant at *p<0.05; **p<0.01; ***p<0.001* | | | | | | | | | | | | | | | | | | | |
| *SD, Standard Deviation; SUD, substance use disorder; AIMS, Access Increases in Mental Health and Substance Abuse Services; SASE, Substance Abuse Service Expansion; HC, health center; HRSA, Health Resources and Services Administration; MAT, Medication Assisted Treatment; CI, confidence interval.* | | | | | | | | | | | | | | | | | | | |
